# Dyslipidemia in Pediatric Type 2 Diabetes Mellitus

**DOI:** 10.1007/s11892-020-01336-6

**Published:** 2020-09-09

**Authors:** Bhuvana Sunil, Ambika P. Ashraf

**Affiliations:** Department of Pediatrics, Division of Endocrinology and Diabetes, University of Alabama at Birmingham, CPPII M30, 1601 4th Ave S, Birmingham, AL 35233 USA

**Keywords:** Dyslipidemia, Insulin resistance, Type 2 diabetes, Cardiovascular risk, Pediatric

## Abstract

**Purpose of Review:**

Cardiovascular (CV) disease is a major cause of mortality in type 2 diabetes mellitus (T2D). Dyslipidemia is prevalent in children with T2D and is a known risk factor for CVD. In this review, we critically examine the epidemiology, pathophysiology, and recommendations for dyslipidemia management in pediatric T2D.

**Recent Findings:**

Dyslipidemia is multifactorial and related to poor glycemic control, insulin resistance, inflammation, and genetic susceptibility. Current guidelines recommend lipid screening after achieving glycemic control and annually thereafter. The desired lipid goals are low-density lipoprotein cholesterol (LDL-C) < 100 mg/dL, high-density lipoprotein cholesterol (HDL-C) > 35 mg/dL, and triglycerides (TG) < 150 mg/dL.

**Summary:**

If LDL-C remains > 130 mg/dL after 6 months, statins are recommended with a treatment goal of < 100 mg/dL. If fasting TG are > 400 mg/dL or non-fasting TG are > 1000 mg/dL, fibrates are recommended. Although abnormal levels of atherogenic TG-rich lipoproteins, apolipoprotein B, and non-HDL-C are commonly present in pediatric T2D, their measurement is not currently considered in risk assessment or management.

## Introduction

Dyslipidemia is highly prevalent in children and adolescents with type 2 diabetes mellitus (T2D). Prominent risk factors including obesity, insulin resistance (IR), hypertension, and sedentary lifestyle tend to cluster in at-risk children. As T2D is an important independent cardiovascular (CV) risk factor, it is essential to recognize and manage dyslipidemia to prevent the anticipated CV morbidity.

The typical dyslipidemia pattern in T2D includes elevated serum triglycerides (TG), decreased high-density lipoprotein cholesterol (HDL-C), and, occasionally, elevated low-density lipoprotein cholesterol (LDL-C) levels [1]. The other less commonly evaluated, non-conventional lipoprotein abnormalities include elevated very low-density lipoprotein cholesterol (VLDL-C), non-HDL-C, small, dense LDL-C, and apolipoprotein B100 (apo B) concentrations [2–5].

## Pathophysiology and Patterns of Dyslipidemia in Type 2 Diabetes

Insulin regulates lipid metabolism and cholesterol homeostasis. Concurrent obesity, metabolic syndrome, and hyperglycemia further worsen the dysregulated lipid metabolism in children and adolescents with T2D. In this section, we review the relevant lipid and lipoprotein abnormalities.

### Dysregulated Triglyceride Metabolism

Circulating TGs are a mixture of TG-rich lipoproteins (TRLPs), i.e., chylomicrons, chylomicron remnants, VLDL-C, and intermediate-density lipoprotein cholesterol (IDL-C), each with varying cholesterol and TG concentrations. Chylomicrons have very little cholesterol content, whereas VLDL-C and IDL-C have substantial amounts of cholesterol.

Studies have shown that in all stages of impaired glucose tolerance including prediabetes, lipoprotein abnormalities can be detected [6]. Early on in T2D, relative insulin deficiency from insulin resistance promotes lipolysis and increased free fatty acid (FFA) flux into the portal circulation. This is exacerbated by the excess consumption of carbohydrates and fats, which triggers de novo lipogenesis. Elevated FFA can stimulate insulin secretion. Chronically elevated FFA in the liver and muscle worsen insulin resistance, ultimately leading to accelerated β-cell destruction and insulin deficiency. The increased FFA flux promotes TG production and hepatic secretion of the TG-rich VLDL-C.

VLDL-C secretion is dependent on TG availability, which is dependent on FFA availability. Despite the milieu of IR, the liver remains sensitive to the effect of insulin on net lipid synthesis [7]. The transcription factor sterol response element-binding protein-1c (SREBP-1c) is considered the major transcriptional factor that regulates hepatic de novo lipogenesis [8]. Elevated insulin levels stimulate VLDL-C and TG secretion by regulating the SREBP-1c, promoting increased TG incorporation and formation of larger VLDL-C particles [9]. Consumption of a carbohydrate-rich diet can stimulate significant insulin secretion in youth which leads to chronic stimulation of VLDL-C secretion. It has been postulated that hyperglycemia also stimulates the formation of larger VLDL-C via the carbohydrate-responsive element-binding protein [10]. Increased apo B production in the liver leads to impaired catabolism of VLDL-C, further increasing the VLDL-C concentration in circulation [11].

The lipases, lipoprotein lipase (LPL) and hepatic lipase, hydrolyze TGs to FFA. Typically, insulin stimulates the activity of LPL. Due to the relative insulin deficiency and/or insulin resistance in T2D, TG hydrolysis is impaired [12]. In general, fasting TG concentrations are usually representative of VLDL-C.

Chylomicrons are produced in the enterocytes from dietary lipids which primarily serve as the transport vehicle for triacyl glycerols, packaged with apoB48. Chylomicrons are typically cleared from circulation within 2 h of food intake by LPL-mediated hydrolysis. Persistence of chylomicrons in the blood following a fast is indicative of dysfunctional LPL activity. At TG concentrations of > 880 mg/dL, chylomicrons predominate compared with VLDL-C. Individuals with multiple predisposing genetic variants have a predisposition to develop hyperchylomicronemia in the presence of clustering of risk factors such as obesity, metabolic syndrome, and T2D [13]. In patients with T2D with elevated fasting hypertriglyceridemia, postprandial TGs are 2–4 times elevated depending on the fat content of the meal. When LPL activity is defective from relative or absolute insulin deficiency in these patients, any additional TG entering the plasma will increase the TG in a non-linear fashion. Furthermore, increased consumption of dietary fat also leads to increased apo B48 production from the enterocytes leading to increased production of chylomicrons. Adults with T2D have also been demonstrated to have elevated Apo C-III levels, promoting hyperchylomicronemia.

It has been proposed that although initially the FFA flux causes increased apo B production [14], when sustained, it may inhibit apo B secretion while at the same time inhibiting apo B degradation [15]. The net effect is an increased availability of apo B resulting in increased VLL-C and non-HDL-C secretion [16, 17]. T2D has additionally shown altered gene expression of proteins including Niemann-Pick C1-Like1 (NPL1L1), ATP-binding cassette protein G5/8, and microsomal triglyceride transfer expression, further contributing to chylomicronemia [18].

An overall decrease in clearance of the TRLPs also contributes to the elevation in serum TG levels. Hydrolysis of TRLPs produces TRL remnants that are typically enriched in cholesterol and apo E but depleted in TG. TRL remnants can contribute to early atherosclerotic lesions either by direct penetration of the arterial walls where the wall is permeable [19] or can activate an inflammatory cascade by elevated FFA and lysolecithin levels [19, 20]. Although LDL-C is considered the most atherogenic component, other apo B-containing lipoproteins, TRL remnants and Lp(a) have now been thought to contribute to intimal cholesterol deposition, principally as they contain a comparable number of cholesterol molecules per particle as LDL-C [21, 22].

### Abnormalities in LDL-C and HDL-C

Patients with T2D tend to have lower HDL-C concentrations partly from obesity and hypertriglyceridemia [23, 24]. Cholesterol ester transfer protein (CETP) mediates the exchange of TGs from VLDL-C and chylomicron remnants to LDL-C and HDL-C. In T2D, enhanced TRLP levels upregulate CETP activity, leading to TG enriched LDL-C and HDL-C [25, 26]. With the enhanced hepatic lipase activity with insulin resistance and lipolysis of TG, smaller dense LDL-C particles are formed. This is apparent in metabolic syndrome, where normal LDL-C levels may be accompanied by the LDL-C particles that are denser [27]. Small dense LDL-C, the so-called pattern B, is known to be more atherogenic. LDL-C atherogenicity is dependent at least in part on its oxidisability, which is increased by hyperglycemia.

Moreover, lower levels of apo A-I and HDL-C levels are seen in T2D. Apo A-I, associated with TG-enriched HDL-C, has lesser affinity for smaller, denser HDL-C [28, 29]. This leads to dissociation of apo A-I and enhanced clearance. There is also evidence to suggest reduced apo A-I formation in patients with T2D, leading to a net lower apo A-I levels [30]. Patients with T2D tend to have lower HDL-C concentrations partly from obesity and hypertriglyceridemia [23, 24]. The MESA study identified that HDL particle number rather than the cholesterol content predicted the functionality [31].

### Apolipoprotein B (Apo B) Changes

There are two Apo B lipoproteins: apo B48 transports the chylomicrons and apo B100 transports the atherogenic lipoproteins such as VLDL-C, IDL-C, and LDL-C. As previously mentioned, dyslipidemia in T2D is characterized by elevated apo B concentration (reduced clearance and increased synthesis). Multiple studies thus far have shown elevated apo B levels in obesity, metabolic syndrome, and T2D, i.e., conditions with mixed dyslipidemia [3, 5, 32–36]. The more conventional use of LDL-C and TG levels may underestimate the significance of the dyslipidemia in children and adolescents with T2D. In the pediatric First Nation children study, in the presence of elevated TG, only 25% had an elevated LDL-C level compared with 37% with an elevated apo B level [32]. Conventional clinical screening and management of dyslipidemia with a traditional lipid profile may not be as sensitive to detail the overall atherogenic risk [37] and underestimate the severity of dyslipidemia in children and adolescents with T2D, detailed lipoprotein analysis including apo B measurements may be indicated in children and adolescents with T2D.

### Elevated Non-HDL Cholesterol

Non-HDL cholesterol (non-HDL-C) can be derived by subtracting HDL-C from total cholesterol, which is a measure of all apo B 100 containing particles (LDL-C, VLDL-C, IDL-C, TRL remnants). Non-HDL-C is relatively unaffected by the non-fasting status and forms a reliable screening measure in children [38]. Since VLDL-C and IDL-C are elevated in T2D, non-HDL-C is usually elevated. In the PDAY study, an increase of 30 mg/dL non-HDL-C was associated with increase in the severity of atherosclerotic lesions [39]. Despite its clinical utility, clear guidelines on the management of non-HDL-C are lacking. In the 2011 National Heart, Lung, and Blood Institute (NHLBI) guidelines for pediatric dyslipidemia, T2D is classified as a high-risk condition and children with non-HDL-C ≥ 145 mg/dL (95th percentile) merit treatment with a goal to bring it down to < 120 mg/dL (< 85th percentile) [40]. According to the NLA, T2D is a high-risk condition for ASCVD, and if a child or adolescent has a non-HDL-C l of ≥ 145 mg/dL, then additional follow-up and management are recommended; however, specific targeted goals for non-HDL-C with T2D have not been established [41]. The ADA and ISPAD guidelines do not specifically incorporate non-HDL-C in the management algorithm [42, 43]. This may represent a source of ambiguity in the recognition and management of elevated non-HDL-C in pediatric T2D.

### Relevance of Lipoprotein(a)

Lipoprotein(a) [Lp(a)] is a highly atherogenic lipoprotein that attaches to the apo B100 moiety of LDL-C particle. Current evidence in adults suggests a link between Lp(a) concentrations and a variety of CV related outcomes [44–48]. Currently, there are no established Lp(a) lowering strategy in youth. The data that support screening for Lp(a) is emerging. Information about a child’s Lp(a) level may help the provider establish a global risk assessment and reiterate the importance of heart-healthy lifestyle to the family, with potential for cascade screening in the future [49].

## Epidemiology and Lipid Trends in Children and Adolescents with T2D

In parallel with rising rates of obesity, the prevalence of T2D in children has continued rising, disproportionately affecting racial and ethnic minorities. In the USA, T2D prevalence in non-Hispanic Whites is 5.5%, and among non-Hispanic Blacks is 37.6% [50].

The SEARCH for Diabetes in Youth Study included 283 children with T2D and showed that 33% of patients with T2D had elevated total cholesterol (TC) levels of > 200 mg/dL [2]. The prevalence of LDL-C > 160 mg/dL in children over the age of 10 years was 9%, and TG > 400 mg/dL was also 9%. Among the older youth, 44% of those with T2D had HDL-C levels < 40 mg/dL. Interestingly, although 24% of those with T2D had a concentration of LDL-C that would warrant pharmacologic intervention if the levels were persistent and non-responsive to diet and lifestyle changes, a very few of those were receiving lipid-lowering therapy, highlighting the need for increased awareness among treating providers [2]. The SEARCH study also illustrated that in youth with T2D who were in poor glycemic control, percentages with high TC, LDL-C, and TG concentrations were 65%, 43%, and 40%, respectively [51].

The Treatment Options for Type 2 Diabetes in Adolescents and Youth (TODAY) study showed that 4.5% had elevated LDL-C levels and 21% of children had elevated TG levels [52]. These children were found to have elevated markers of inflammation, indicating a favorable milieu for development of atherosclerosis [52]. Worsening glycemic control was known to be a significant factor in the worsening of dyslipidemia during follow-up of this cohort of patients [53].

Racially stratified data are rare in pediatric T2D. Increased total cholesterol, LDL-C, TG, and decreased HDL-C have been reported in a small cohort of African American youth with T2D [54]. In a study of First Nation children with T2D, the mean TC, TG, LDL-C, total cholesterol/HDL-C ratio, and apo B were all significantly higher, and HDL-C was significantly lower compared with racially matched controls [32]. Ethnic-specific differences seen in the general population were also reflected in the TODAY cohort, with lower TG levels observed in non-Hispanic Blacks compared with both Hispanic and non-Hispanic Whites [52].

Data on lipoprotein subclasses in pediatric individuals with T2D are limited. Table 1 depicts data from recent studies emphasizing lipoprotein subclass patterns in children with varying spectra of insulin resistance related conditions, obesity, and metabolic syndrome with prediabetes to overt T2D.

## Current Screening Guidelines for Dyslipidemia in Children and Adolescents with T2D

The American Diabetes Association (ADA) and American Academy of Pediatrics (AAP) have recommended for screening of dyslipidemia in youth with new onset T2D once glycemic control is established or after 3 months after medication initiation and annually thereafter [42, 43]. The International Society for Pediatric and Adolescent and Adolescent Diabetes (ISPAD) also recommends lipid screening at diagnosis; repeating testing for dyslipidemia once glycemic control has been achieved or after 3 months of initiation of medication and annually thereafter [43]. If the lipid profile is normal, it is also recommended to screen with a fasting lipid profile annually thereafter [42].

## Current Goals and Recommendations for Management of Dyslipidemia in Children and Adolescents with T2D

### Treatment of Elevated LDL-C

Optimal goals of lipid levels are LDL-C < 100 mg/dL, HDL-C > 35 mg/dL, and TG < 150 mg/dL [42, 43]. If the lipids are abnormal, the first step is to optimize the glycemic control as best as possible with lifestyle modifications and incorporation of medical nutrition therapy (MNT). The MNT should include limiting calories from fat to 25–30%, saturated fat to < 7%, cholesterol < 200 mg/day, avoiding *trans* fats, and aiming for ∼ 10% calories from monounsaturated fats for elevated LDL-C (similar to the American Heart Association step 2 diet).

If LDL-C remains > 130 mg/dL after 6 months of dietary intervention and optimized glycemic control, statin therapy, with a treatment goal of LDL-C < 100 mg/dL, is indicated in children over 8 years of age [42]. In practice, we also screen for other secondary causes of dyslipidemia including thyroid function studies prior to starting treatment. Other suggested serum testing prior to initiation of statin therapy includes serum albumin, renal function testing, and a pregnancy screen as deemed clinically necessary. Liver function studies and serum creatinine kinase levels are useful to monitor for future potential adverse effects. It is important to counsel on the potential teratogenicity of statins prior to initiation of therapy and encourage contraception for females who are sexually active [59].

Since T2D is a high-risk category ASCVD risk factor, the use of a high potency, maximally effective, and well-tolerated dose of statin is optimal. The maximum daily doses studied in pediatrics for the various available statins are 40 mg for lovastatin, pravastatin, and simvastatin; 20 mg for atorvastatin and rosuvastatin; and 80 mg for fluvastatin. Atorvastatin and rosuvastatin are recommended as first line in adult guidelines because of RCTs demonstrating their efficiency in lowering the ASCVD risk [60]. There is significant evidence from adult trials that lowering the LDL-C by a statin reduces the CV risk [61].

Studies in children with familial hypercholesterolemia have established that statins are effective and safe in children [62]. The risk of incident T2D with statin use has been debated. The proposed mechanisms include impaired insulin secretion, increased insulin resistance, and increased glucose secretion [63, 64]. In pediatrics, the available data has been mostly reassuring [65–67]. Though, the data may underrepresent the racial and ethnic minorities in whom the prevalence of T2D is higher. With the current knowledge, given the overall accelerated increased atherogenic risk with dyslipidemia and T2D from a young age in children with T2D, the benefits of statin therapy outweigh any potential risks.

### Treatment of Elevated Triglycerides

For elevated TGs, weight reduction, decreasing simple carbohydrates, and increasing dietary n-3 fatty acids are recommended. [68]

#### TG > 400 mg/dL

If the fasting TGs are > 400 mg/dL or non-fasting TGs are > 1000 mg/dL, there is a substantial increase in the risk of pancreatitis [69]. In addition to optimization of glycemic control, it is recommended to begin fibrate therapy, to achieve a goal of < 400 mg/dL [42]. Fibrates act through PPAR-α and downregulate apo CIII, leading to reduced TG concentrations.

#### TG < 400 mg/dL

Even though we do not have adequate pediatric data on the independent role of TGs on atherosclerotic CV disease, both fasting and post-hypertriglyceridemia are recognized as significant CV risk factors. While fasting hypertriglyceridemia is mostly from elevated circulating VLDL-C levels, postprandial hypertriglyceridemia is from VLDL-C, IDL-C, and TRL remnant particles. The current ADA recommendation does not address the management of hypertriglyceridemia up to 400 mg/dL for CV risk reduction in these children, even though the 2011 NHLBI recommends statin treatment for persistently elevated non-HDL-C >145 mg/dL in children with mixed dyslipidemia [70–72].

It is important to concomitantly manage coexistent obesity and IR. Weight loss is recommended using a multimodal and graduated approach incorporating lifestyle changes, increasing physical activity to ≥ 5 h/week of moderate to vigorous physical activity [73], pharmacotherapy, and consideration of bariatric surgery [74].

#### Severe Hypertriglyceridemia (TG > 1000 mg/dL)

Severe hypertriglyceridemia in patients with uncontrolled diabetes and those with previous episodes of pancreatitis require inpatient admission and intensive monitoring when the TG levels are higher than 2000 mg/dL. These levels may also be seen at diagnosis or anytime during the course of T2D. In our practice, asymptomatic hypertriglyceridemia between 1000–2000 mg/dL is treated with fat-free diet along with aggressive management of hyperglycemia with insulin until the serum TG concentration is < 1000 mg/dL. 

At TG concentrations > 2000 mg/dL, nothing by mouth (NPO), maintenance intravenous hydration (IVF) and insulin drip at 0.05–0.1 U/kg/h are beneficial even in patients without diabetic ketoacidosis [75–77], as this helps in the rapid decline of TG levels. We titrate insulin drip to maintain blood glucoses between 100 and 200 mg/dL and initiate dextrose infusions to prevent hypoglycemia. Ensuring strict glycemic control is indicated [42]. Insulin therapy has a dual benefit of controlling hyperglycemia and hypertriglyceridemia in the setting of T2D.

Intravenous regular insulin therapy activates endothelial LPL and enhances degradation and clearance of TG from the circulation, and insulin inhibits hormone-sensitive lipase in adipocytes, preventing TG and FFA release from the adipose tissue [78]. Plasmapheresis is used as an option to reduce serum TG levels rapidly and is reserved for symptomatic patients with severe hypertriglyceridemia and pancreatitis, end organ failure, or shock.

### Effect of Concurrent Therapeutic Strategies for T2D on Dyslipidemia

There is emerging data on additional therapeutic strategies for the management of pediatric T2D. Table 2 provides a summary of some of the important studies that have evaluated the effect of these therapeutic options on dyslipidemia.

**Table 2:** Effect of current therapeutic strategies on dyslipidemia in type 2 diabetes in children

Medication/therapeutic strategy (references)	TG/VLDL-C	HDL-C	LDL-C	Comments
Low-carbohydrate diet [79–81]	↓	↑	↔/↑	Mostly cohort studies with short term data
Mediterranean diet [82, 83]	↓	↑	↓	Mostly cohort studies with short term data
Insulin [52, 53, 84]	↓	↑	↔	Significant when A1C ≤ 8%
Metformin [52, 53, 84, 85]	↓	Mild ↑	↓	TODAY study: 55.9% of the youth remained at the LDL-C goal of < 100 mg/dL over the first 3 years. Levels of TG, apo B, and non-HDL-C rose from baseline to the end of the first year and remained at a higher level for the next 2 years. Only improved glycemic control and weight loss have been associated with improvement in lipid levels
Liraglutide [86, 87]	↓	↔	↔	No differences were apparent in longer term studies. Unclear if the results due mostly normal lipids at baseline/limited sample size
Bariatric surgery [88–92]	↓	↑	Mild ↓	Minor complications were higher in adolescent studies.

*TG* triglyceride, *VLDL-C* very low-density lipoprotein cholesterol, *HDL-C* high-density lipoprotien cholesterol, *LDL-C* low-density lipoprotein cholesterol

**Table 1:** Lipoprotein subclass patterns in children with varying spectra of insulin resistance related conditions

Lipoprotein pattern	Method of analysis	Number and characteristics of participants	Vascular indices and other outcomes	Comments (Ref)
↓phospholipid content in large HDL-C ↓ HDL-C ↓ apoAI, apoE, apoCI, paraoxonase in large HDL-C overall ↓ large HDL-C	Gel filtration (size exclusion) chromatography, mass spectrometry, and proteomic analysis	10 children with T2D	Negative correlation to pulse wave velocity	Only males included [55]
↑ small LDL-C ↑ small HDL-C ↓ large HDL-C and ↑ small HDL-C	Nuclear magnetic resonance spectroscopy	21 children with prediabetes	NA	Obese prediabetic adolescents have a significantly more atherogenic lipoprotein profile compared with obese normoglycemic peers [56]
↑ small LDL-C ↑ small HDL-C and large VLDL-C Overall smaller LDL-C and HDL-C	Nuclear magnetic resonance spectroscopy	194 children with varying spectra of IR	NA	Race stratified: Black male children had smaller VLDL-C, and Black female children had larger HDL-C size [57]
↑ small dense LDL-C ↑ apo B ↓total HDL-C, HDL-2, and HDL-3	Vertical autoprofile lipoprotein analysis	77 children with T2D	NA	Retrospective review in which poor glycemic control was associated with abnormal lipoprotein profiles in both T1D and T2D [58]
↑ LDL-C, non-HDL-C, apoB LDL pattern B (↑ small dense LDL-C)	Vertical autoprofile lipoprotein analysis	93 children with T2D of which 67% with HbA1C >8%	NA	Retrospective review of mostly female, mostly African American children [5]
↑ LDL-C and ↓ LDL-C size ↑ VLDL-C particle size and number ↓ HDL-C size	Nuclear magnetic resonance spectroscopy	214 children with T2D	LDL-P was the most consistent contributor (to the carotid bulb and internal carotid intima media thickness, augmentation index)	Lean, obese, and children with T2D are compared.Children with T2D had the largest number of the smallest and most dense LDL-C particles. [37]

*LDL-C* low-density lipoprotein cholesterol, *HDL-C* high-density lipoprotien cholesterol, *VLDL-C* very low-density lipoprotein cholesterol, *IR* insulin resistance, *T2D* type 2 diabetes mellitus, *T1D* type 1 diabetes mellitus

### Ongoing Clinical Studies for Dyslipidemia in T2D

There is some data in adults that antisense-mediated lowering of apo CIII by volanesorsen improves dyslipidemia and insulin sensitivity in T2D [93]―this is yet to be studied in children with T2D. In adults 18 years and older, pemafibrate, a selective PPAR modulator that reduces TG levels by 35–45%, is being evaluated in PROMINENT (Pemafibrate to Reduce Cardiovascular Outcomes by Reducing Triglycerides in Patients with Diabetes), in a phase 3 ASCVD outcomes study of ∼ 10,000 patients with T2D and HTG (NCT03071692). A phase 2 trial of IONIS-ANGPTL3-L_Rx_ (this acts primarily within hepatocytes to block production of hepatic ANGPTL3) in patients with T2D, hepatosteatosis, and TG > 200 mg/dL is underway (NCT03371355) in adults. Some of the ongoing clinical trials aim to test newer medications whose primary outcome is improving glycemic control in pediatric patients, with dyslipidemia being studied as a secondary outcome of the study get another ongoing studies include clinical trials on the safety and efficacy of ertugliflosin, an SGLT-2 inhibitor (NCT04029480), a DPP-IV inhibitor, sitagliptin (NCT01760447), and a GLP-1 analog, albiglutide (NCT03015519).

## Conclusions

Dyslipidemia is exceedingly common in children and adolescents with T2D. In the presence of T2D in a metabolically unhealthy child with obesity, there is clustering of several CV risk factors, compounding the risk of morbidity and mortality in adulthood. Current guidelines for management of dyslipidemia in T2D do not consider non-HDL-C, apo B, small dense LDL-C, and triglyceride rich lipoproteins. Studies are warranted to further elucidate the accurate risk assessment and optimal therapies in the management of dyslipidemia in pediatric T2D.

## Abbreviations

T2D: Type 2 diabetes mellitus
IR: Insulin resistance
CV: Cardiovascular
TC: Total cholesterol
TG: Triglyceride
HDL-C: High-density lipoprotein cholesterol
LDL-C: Low-density lipoprotein cholesterol
VLDL-C: Very low-density lipoprotein cholesterol
IDL-C: Intermediate-density lipoprotein cholesterol
apo B: Apolipoprotein B100
TRLP: Triglyceride-rich lipoprotein
NHLBI: National Heart, Lung, and Blood Institute
NLA: National Lipid Association
ADA: The American Diabetes Association
ISPAD: International Society for Pediatric and Adolescent Diabetes
ASCVD: Atherosclerotic cardiovascular disease

## References

[1] Mazzone T, Chait A, Plutzky J (2008). Cardiovascular disease risk in type 2 diabetes mellitus: insights from mechanistic studies. Lancet..

[2] Kershnar AK, Daniels SR, Imperatore G, Palla SL, Petitti DB, Pettitt DJ (2006). Lipid abnormalities are prevalent in youth with type 1 and type 2 diabetes: the SEARCH for diabetes in youth study. J Pediatr.

[3] Albers JJ, Marcovina SM, Imperatore G, Snively BM, Stafford J, Fujimoto WY (2008). Prevalence and determinants of elevated apolipoprotein B and dense low-density lipoprotein in youths with type 1 and type 2 diabetes. J Clin Endocrinol Metab.

[4] Hamman RF, Bell RA, Dabelea D, D'Agostino RB, Dolan L, Imperatore G (2014). The SEARCH for diabetes in youth study: rationale, findings, and future directions. Diabetes Care.

[5] Pelham JH, Hanks L, Aslibekyan S, Dowla S, Ashraf AP (2019). Higher hemoglobin A1C and atherogenic lipoprotein profiles in children and adolescents with type 2 diabetes mellitus. J Clin Translat Endocrinol.

[6] Wang J, Stančáková A, Soininen P, Kangas A, Paananen J, Kuusisto J (2012). Lipoprotein subclass profiles in individuals with varying degrees of glucose tolerance: a population-based study of 9399 Finnish men. J Intern Med.

[7] Chait A, Ginsberg HN, Vaisar T, Heinecke JW, Goldberg IJ, Bornfeldt KE (2020). Remnants of the triglyceride-rich lipoproteins, diabetes, and cardiovascular disease. Diabetes..

[8] Ferré P, Foufelle F (2007). SREBP-1c transcription factor and lipid homeostasis: clinical perspective. Hormone Res Paediatr.

[9] Yamashita T, Eto K, Okazaki Y, Yamashita S, Yamauchi T, Sekine N (2004). Role of uncoupling protein-2 up-regulation and triglyceride accumulation in impaired glucose-stimulated insulin secretion in a β-cell lipotoxicity model overexpressing sterol regulatory element-binding protein-1c. Endocrinology..

[10] Wang Y, Viscarra J, Kim SJ, Sul HS (2015). Transcriptional regulation of hepatic lipogenesis. Nat Rev Mol Cell Biol.

[11] Mamo JC, Szeto L, Steiner G (1990). Glycation of very low density lipoprotein from rat plasma impairs its catabolism. Diabetologia..

[12] Boden G, Laakso M (2004). Lipids and glucose in type 2 diabetes: what is the cause and effect?. Diabetes Care.

[13] Brahm AJ, Hegele RA (2015). Chylomicronaemia—current diagnosis and future therapies. Nat Rev Endocrinol.

[14] Dixon JL, Ginsberg HN (1993). Regulation of hepatic secretion of apolipoprotein B-containing lipoproteins: information obtained from cultured liver cells. J Lipid Res.

[15] Zhang YL, Hernandez-Ono A, Ko C, Yasunaga K, Huang LS, Ginsberg HN (2004). Regulation of hepatic apolipoprotein B-lipoprotein assembly and secretion by the availability of fatty acids. I. Differential response to the delivery of fatty acids via albumin or remnant-like emulsion particles. J Biol Chem.

[16] Lewis GF, Carpentier A, Adeli K, Giacca A (2002). Disordered fat storage and mobilization in the pathogenesis of insulin resistance and type 2 diabetes. Endocr Rev.

[17] Xiao C, Dash S, Morgantini C, Lewis GF (2014). New and emerging regulators of intestinal lipoprotein secretion. Atherosclerosis..

[18] Lally S, Tan C, Owens D, Tomkin G (2006). Messenger RNA levels of genes involved in dysregulation of postprandial lipoproteins in type 2 diabetes: the role of Niemann–Pick C1-like 1, ATP-binding cassette, transporters G5 and G8, and of microsomal triglyceride transfer protein. Diabetologia..

[19] Ginsberg HN (2002). New perspectives on atherogenesis: role of abnormal triglyceride-rich lipoprotein metabolism. Circulation..

[20] Twickler TB, Dallinga-Thie GM, Cohn JS, Chapman MJ (2004). Elevated remnant-like particle cholesterol concentration: a characteristic feature of the atherogenic lipoprotein phenotype. Circulation..

[21] Toth PP (2016). Triglyceride-rich lipoproteins as a causal factor for cardiovascular disease. Vasc Health Risk Manag.

[22] Nordestgaard BG (2016). Triglyceride-rich lipoproteins and atherosclerotic cardiovascular disease: new insights from epidemiology, genetics, and biology. Circ Res.

[23] Sun JT, Liu Y, Lu L, Liu HJ, Shen WF, Yang K (2016). Diabetes-invoked high-density lipoprotein and its association with coronary artery disease in patients with type 2 diabetes mellitus. Am J Cardiol.

[24] Rosso LG, Lhomme M, Meroño T, Dellepiane A, Sorroche P, Hedjazi L (2017). Poor glycemic control in type 2 diabetes enhances functional and compositional alterations of small, dense HDL3c. Biochimica et Biophysica Acta (BBA).

[25] Bouillet B, Gautier T, Blache D, de Barros J-PP, Duvillard L, Petit J-M (2014). Glycation of apolipoprotein C1 impairs its CETP inhibitory property: pathophysiological relevance in patients with type 1 and type 2 diabetes. Diabetes Care.

[26] Morton RE, Zilversmit D (1983). Inter-relationship of lipids transferred by the lipid-transfer protein isolated from human lipoprotein-deficient plasma. J Biol Chem.

[27] Kathiresan S, Otvos JD, Sullivan LM, Keyes MJ, Schaefer EJ, Wilson PW (2006). Increased small low-density lipoprotein particle number: a prominent feature of the metabolic syndrome in the Framingham heart study. Circulation..

[28] Gordon SM, Deng J, Lu LJ, Davidson WS (2010). Proteomic characterization of human plasma high density lipoprotein fractionated by gel filtration chromatography. J Proteome Res.

[29] Baker HN, Delahunty T, Gotto AM, Jackson RL (1974). The primary structure of high density apolipoprotein-glutamine-I. Proc Natl Acad Sci.

[30] Brahimaj A, Ligthart S, Ikram MA, Hofman A, Franco OH, Sijbrands EJG (2017). Serum levels of apolipoproteins and incident type 2 diabetes: a prospective cohort study. Diabetes Care.

[31] Tehrani DM, Zhao Y, Blaha MJ, Mora S, Mackey RH, Michos ED (2016). Discordance of low-density lipoprotein and high-density lipoprotein cholesterol particle versus cholesterol concentration for the prediction of cardiovascular disease in patients with metabolic syndrome and diabetes mellitus (from the multi-ethnic study of atherosclerosis [MESA]). Am J Cardiol.

[32] Sellers EA, Yung G, Dean HJ (2007). Dyslipidemia and other cardiovascular risk factors in a Canadian first nation pediatric population with type 2 diabetes mellitus. Pediatr Diabetes.

[33] Hannon TS, Arslanian SA. The changing face of diabetes in youth: lessons learned from studies of type 2 diabetes. Ann N Y Acad Sci. 2015;(10)1353(1):113–7. 10.1111/nyas.12939 26448515

[34] Sellers EA, Singh GR, Sayers SM (2009). Apo-B/AI ratio identifies cardiovascular risk in childhood: the Australian aboriginal birth cohort study. Diab Vasc Dis Res.

[35] Azad K, Parkin J, Laker M, Alberti K (1994). Circulating lipids and glycaemic control in insulin dependent diabetic children. Arch Dis Child.

[36] West NA, Hamman RF, Mayer-Davis EJ, D'Agostino RB, Marcovina SM, Liese AD (2009). Cardiovascular risk factors among youth with and without type 2 diabetes: differences and possible mechanisms. Diabetes Care.

[37] Urbina EM, McCoy CE, Gao Z, Khoury PR, Shah AS, Dolan LM (2017). Lipoprotein particle number and size predict vascular structure and function better than traditional lipids in adolescents and young adults. J Clin Lipidol.

[38] Miyazaki A, Oguri A, Ichida F (2016). Non-high-density lipoprotein cholesterol as a cardiovascular risk screening tool in children. Pediatr Int.

[39] Strong JP, Malcom GT, McMahan CA, Tracy RE, Newman WP, Herderick EE (1999). Prevalence and extent of atherosclerosis in adolescents and young adults: implications for prevention from the pathobiological determinants of atherosclerosis in youth study. Jama..

[40] FOR EPOIG, CHILDREN RRI (2011). Expert panel on integrated guidelines for cardiovascular health and risk reduction in children and adolescents: summary report. Pediatrics..

[41] Bays HE, Jones PH, Orringer CE, Brown WV, Jacobson TA (2016). National lipid association annual summary of clinical lipidology 2016. J Clin Lipidol.

[42] Association AD (2020). 13 Children and adolescents: standards of medical care in diabetes− 2020. Diabetes Care.

[43] Zeitler P, Arslanian S, Fu J, Pinhas-Hamiel O, Reinehr T, Tandon N (2018). ISPAD clinical practice consensus guidelines 2018: type 2 diabetes mellitus in youth. Pediatr Diabetes.

[44] Paré G, Çaku A, McQueen M, Anand SS, Enas E, Clarke R (2019). Lipoprotein (a) levels and the risk of myocardial infarction among 7 ethnic groups. Circulation..

[45] Emerging Risk Factors C, Erqou S, Kaptoge S, Perry PL, Di Angelantonio E, Thompson A (2009). Lipoprotein(a) concentration and the risk of coronary heart disease, stroke, and nonvascular mortality. JAMA..

[46] Nordestgaard BG, Chapman MJ, Ray K, Borén J, Andreotti F, Watts GF (2010). Lipoprotein (a) as a cardiovascular risk factor: current status. Eur Heart J.

[47] Kronenberg F, Utermann G (2013). Lipoprotein (a): resurrected by genetics. J Intern Med.

[48] Tsimikas S (2017). A test in context: lipoprotein (a): diagnosis, prognosis, controversies, and emerging therapies. J Am Coll Cardiol.

[49] Wilson DP, Jacobson TA, Jones PH, Koschinsky ML, McNeal CJ, Nordestgaard BG (2019). Use of lipoprotein (a) in clinical practice: a biomarker whose time has come. A scientific statement from the National Lipid Association. J Clin Lipidol.

[50] Jensen ET, Dabelea D (2018). Type 2 diabetes in youth: new lessons from the SEARCH study. Curr Diab Rep.

[51] Petitti DB, Imperatore G, Palla SL, Daniels SR, Dolan LM, Kershnar AK (2007). Serum lipids and glucose control: the SEARCH for diabetes in youth study. Arch Pediatr Adolesc Med.

[52] Lipid and inflammatory cardiovascular risk worsens over 3 years in youth with type 2 diabetes: the TODAY clinical trial. Diabetes Care. 2013;36(6):1758–64. 10.2337/dc12-2388 PMC3661790 23704675

[53] Levitt Katz LE, Bacha F, Gidding SS, Weinstock RS, El Ghormli L, Libman I (2018). Lipid profiles, inflammatory markers, and insulin therapy in youth with type 2 diabetes. J Pediatr.

[54] Taha D (2002). Hyperlipidemia in children with type 2 diabetes mellitus. J Pediatr Endocrinol Metab.

[55] Gordon SM, Davidson WS, Urbina EM, Dolan LM, Heink A, Zang H (2013). The effects of type 2 diabetes on lipoprotein composition and arterial stiffness in male youth. Diabetes..

[56] Magge SN, Prasad D, Koren D, Gallagher PR, Mohler ER, Stettler N (2012). Prediabetic obese adolescents have a more atherogenic lipoprotein profile compared with normoglycemic obese peers. J Pediatr.

[57] Burns SF, Lee S, Arslanian SA (2009). In vivo insulin sensitivity and lipoprotein particle size and concentration in black and white children. Diabetes Care.

[58] Hanks LJ, Pelham JH, Vaid S, Casazza K, Ashraf AP (2016). Overweight adolescents with type 2 diabetes have significantly higher lipoprotein abnormalities than those with type 1 diabetes. Diabetes Res Clin Pract.

[59] Newman CB, Preiss D, Tobert JA, Jacobson TA, Page RL, Goldstein LB (2019). Statin safety and associated adverse events: a scientific statement from the American Heart Association. Arterioscler Thromb Vasc Biol.

[60] Grundy SM, Stone NJ, Bailey AL, Beam C, Birtcher KK, Blumenthal RS (2019). 2018 AHA/ACC/AACVPR/AAPA/ABC/ACPM/ADA/AGS/APhA/ASPC/NLA/PCNA guideline on the management of blood cholesterol: a report of the American College of Cardiology/American Heart Association task force on clinical practice guidelines. Circulation..

[61] Colhoun H, Thomason M, Mackness M, Maton S, Betteridge D, Durrington P (2002). Design of the collaborative AtoRvastatin diabetes study (CARDS) in patients with type 2 diabetes. Diabet Med.

[62] Avis HJ, Hutten BA, Gagné C, Langslet G, McCrindle BW, Wiegman A (2010). Efficacy and safety of rosuvastatin therapy for children with familial hypercholesterolemia. J Am Coll Cardiol.

[63] Lotta LA, Sharp SJ, Burgess S, Perry JRB, Stewart ID, Willems SM (2016). Association between low-density lipoprotein cholesterol-lowering genetic variants and Risk of type 2 diabetes: a meta-analysis. Jama..

[64] Swerdlow DI, Preiss D, Kuchenbaecker KB, Holmes MV, Engmann JE, Shah T (2015). HMG-coenzyme a reductase inhibition, type 2 diabetes, and bodyweight: evidence from genetic analysis and randomised trials. Lancet..

[65] Kusters DM, Avis HJ, de Groot E, Wijburg FA, Kastelein JJ, Wiegman A (2014). Ten-year follow-up after initiation of statin therapy in children with familial hypercholesterolemia. Jama..

[66] Luirink IK, Wiegman A, Kusters DM, Hof MH, Groothoff JW, de Groot E (2019). 20-year follow-up of statins in children with familial hypercholesterolemia. N Engl J Med.

[67] Joyce NR, Zachariah JP, Eaton CB, Trivedi AN, Wellenius GA (2017). Statin use and the risk of type 2 diabetes mellitus in children and adolescents. Acad Pediatr.

[68] Aikenhead A, Lobstein T, Knai C (2011). Review of current guidelines on adolescent bariatric surgery. Clin Obes.

[69] Berglund L, Brunzell JD, Goldberg AC, Goldberg IJ, Sacks F, Murad MH (2012). Evaluation and treatment of hypertriglyceridemia: an endocrine society clinical practice guideline. J Clin Endocrinol Metab.

[70] Murad MH, Hazem A, Coto-Yglesias F, Dzyubak S, Gupta S, Bancos I (2012). The association of hypertriglyceridemia with cardiovascular events and pancreatitis: a systematic review and meta-analysis. BMC Endocr Disord.

[71] Iso H, Naito Y, Sato S, Kitamura A, Okamura T, Sankai T (2001). Serum triglycerides and risk of coronary heart disease among Japanese men and women. Am J Epidemiol.

[72] Nordestgaard BG, Benn M, Schnohr P, Tybjærg-Hansen A (2007). Nonfasting triglycerides and risk of myocardial infarction, ischemic heart disease, and death in men and women. Jama..

[73] Cugnetto ML, Saab PG, Llabre MM, Goldberg R, McCalla JR, Schneiderman N (2008). Lifestyle factors, body mass index, and lipid profile in adolescents. J Pediatr Psychol.

[74] Styne DM, Arslanian SA, Connor EL, Farooqi IS, Murad MH, Silverstein JH (2017). Pediatric obesity-assessment, treatment, and prevention: an endocrine society clinical practice guideline. J Clin Endocrinol Metab.

[75] Jabbar MA, Zuhri-Yafi MI, Larrea J (1998). Insulin therapy for a non-diabetic patient with severe hypertriglyceridemia. J Am Coll Nutr.

[76] Mikhail N, Trivedi K, Page C, Wali S, Cope D (2005). Treatment of severe hypertriglyceridemia in nondiabetic patients with insulin. Am J Emerg Med.

[77] Rawla P, Sunkara T, Thandra KC, Gaduputi V (2018). Hypertriglyceridemia-induced pancreatitis: updated review of current treatment and preventive strategies. Clin J Gastroenterol.

[78] Eckel RH (1989). Lipoprotein lipase. A multifunctional enzyme relevant to common metabolic diseases. N Engl J Med.

[79] Demol S, Yackobovitch-Gavan M, Shalitin S, Nagelberg N, Gillon-Keren M, Phillip M (2009). Low-carbohydrate (low & high-fat) versus high-carbohydrate low-fat diets in the treatment of obesity in adolescents. Acta Paediatr.

[80] Gow ML, Ho M, Burrows TL, Baur LA, Stewart L, Hutchesson MJ (2014). Impact of dietary macronutrient distribution on BMI and cardiometabolic outcomes in overweight and obese children and adolescents: a systematic review. Nutr Rev.

[81] Ebbeling CB, Feldman HA, Chomitz VR, Antonelli TA, Gortmaker SL, Osganian SK (2012). A randomized trial of sugar-sweetened beverages and adolescent body weight. N Engl J Med.

[82] Velázquez-López L, Santiago-Díaz G, Nava-Hernández J, Muñoz-Torres AV, Medina-Bravo P, Torres-Tamayo M (2014). Mediterranean-style diet reduces metabolic syndrome components in obese children and adolescents with obesity. BMC Pediatr.

[83] Giannini C, Diesse L, D'adamo E, Chiavaroli V, de Giorgis T, Di Iorio C (2014). Influence of the Mediterranean diet on carotid intima–media thickness in hypercholesterolaemic children: a 12-month intervention study. Nutr Metab Cardiovasc Dis.

[84] Barr MM, Aslibekyan S, Ashraf AP. Glycemic control and lipid outcomes in children and adolescents with type 2 diabetes. PLoS One 752 One. 2019;14(7):e0219144. 10.1371/journal.pone.0219144 PMC6602203 31260475

[85] Luong DQ, Oster R, Ashraf AP (2015). Metformin treatment improves weight and dyslipidemia in children with metabolic syndrome. J Pediatr Endocrinol Metab.

[86] Kelly AS, Auerbach P, Barrientos-Perez M, Gies I, Hale PM, Marcus C, et al. A randomized, controlled trial of liraglutide for 758 adolescents with obesity. N Engl J Med. 2020;382(22):2117–28. 10.1056/NEJMoa1916038 32233338

[87] Tamborlane WV, Barrientos-Perez M, Fainberg U, Frimer-Larsen H, Hafez M, Hale PM (2019). Liraglutide in children and adolescents with type 2 diabetes. N Engl J Med.

[88] Alqahtani A, Alamri H, Elahmedi M, Mohammed R (2012). Laparoscopic sleeve gastrectomy in adult and pediatric obese patients: a comparative study. Surg Endosc.

[89] Inge TH, Jenkins TM, Xanthakos SA, Dixon JB, Daniels SR, Zeller MH (2017). Long-term outcomes of bariatric surgery in adolescents with severe obesity (FABS-5+): a prospective follow-up analysis. Lancet Diabetes Endocrinol.

[90] Inge TH, Zeller MH, Jenkins TM, Helmrath M, Brandt ML, Michalsky MP (2014). Perioperative outcomes of adolescents undergoing bariatric surgery: the teen-longitudinal assessment of bariatric surgery (teen-LABS) study. JAMA Pediatr.

[91] Olbers T, Beamish AJ, Gronowitz E, Flodmark CE, Dahlgren J, Bruze G (2017). Laparoscopic Roux-en-Y gastric bypass in adolescents with severe obesity (AMOS): a prospective, 5-year, Swedish nationwide study. Lancet Diabetes Endocrinol.

[92] Beamish AJ, Reinehr T (2017). Should bariatric surgery be performed in adolescents?. Eur J Endocrinol.

[93] Yang X, Lee S-R, Choi Y-S, Alexander VJ, Digenio A, Yang Q (2016). Reduction in lipoprotein-associated apoC-III levels following volanesorsen therapy: phase 2 randomized trial results. J Lipid Res.

